# The effects of Cognitive Bias Modification training and oxytocin administration on trust in maternal support: study protocol for a randomized controlled trial

**DOI:** 10.1186/s13063-017-2077-2

**Published:** 2017-07-14

**Authors:** Martine W. F. T. Verhees, Eva Ceulemans, Marian J. Bakermans-Kranenburg, Marinus H. van IJzendoorn, Simon de Winter, Guy Bosmans

**Affiliations:** 1 Parenting and Special Education Research Unit, KU Leuven, Leopold Vanderkelenstraat 32, 3000 Leuven, Belgium; 2 Quantitative Psychology and Individual Differences Research Unit, KU Leuven, Tiensestraat 102, 3000 Leuven, Belgium; 3 Centre for Child and Family Studies, Leiden University, Wassenaarseweg 52, 2333 AK Leiden, The Netherlands

**Keywords:** Trust, Oxytocin, Cognitive Bias Modification (CBM), Interpretation bias, Middle childhood, Attachment, Randomized Controlled Trial (RCT)

## Abstract

**Background:**

Lack of trust in parental support is a transdiagnostic risk factor for the development of psychological problems throughout the lifespan. Research suggests that children’s cognitive attachment representations and related information processing biases could be an important target for interventions aiming to build trust in the parent-child relationship. A paradigm that can alter these biases and increase trust is that of Cognitive Bias Modification (CBM), during which a target processing bias is systematically trained. Trust-related CBM training effects could possibly be enhanced by oxytocin, a neuropeptide that has been proposed to play an important role in social information processing and social relationships. The present article describes the study protocol for a double-blind randomized controlled trial (RCT) aimed at testing the individual and combined effects of CBM training and oxytocin administration on trust in maternal support.

**Methods/design:**

One hundred children (aged 8–12 years) are randomly assigned to one of four intervention conditions. Participants inhale a nasal spray that either contains oxytocin (OT) or a placebo. Additionally, they receive either a CBM training aimed at positively modifying trust-related information processing bias or a neutral placebo training aimed to have no trust-related effects. Main and interaction effects of the interventions are assessed on three levels of trust-related outcome measures: trust-related interpretation bias; self-reported trust; and mother-child interactional behavior. Importantly, side-effects of a single administration of OT in middle childhood are monitored closely to provide further information on the safety of OT administration in this age group.

**Discussion:**

The present RCT is the first study to combine CBM training with oxytocin to test for individual and combined effects on trust in mother. If effective, CBM training and oxytocin could be easily applicable and nonintrusive additions to interventions that target trust in the context of the parent-child relationship.

**Trial registration:**

ClinicalTrials.gov, ID: NCT02737254. Registered on 23 March 2016.

**Electronic supplementary material:**

The online version of this article (doi:10.1186/s13063-017-2077-2) contains supplementary material, which is available to authorized users.

## Background

Lack of trust in parental support puts children at risk for the development of psychological problems across the lifespan [1, 2]. Bowlby [1] proposed that whether children are able to develop trust in parental support depends for a significant part on children’s experiences of care in response to distress during interactions with their parents. Through repeated interactions with sensitive and responsive parenting, children develop trust in the parent and become securely attached [3]. Conversely, when children experience recurrent insensitive, unresponsive or inconsistent parenting, they will have difficulty to develop trust in the parent and become insecurely attached [3]. Research suggests that up to 40% of the children are insecurely attached [4], and although insecure attachment is neither a necessary nor a sufficient cause for psychopathology development, it is considered an important transdiagnostic risk factor [5–7].

Given the risks associated with insecure attachment, there is need for effective interventions that can promote trust in the context of the parent-child relationship [8, 9]. The present study aims to investigate the individual and combined effects of a cognitive and a pharmacological intervention, specifically Cognitive Bias Modification (CBM) training and intranasal oxytocin (OT), on trust in maternal support. Although trust is important for psychological wellbeing across the lifespan, in the current study we focus on middle childhood (age range 8–12 years). Middle childhood has been proposed to be an important period for cognitive trust development, during which trust-related expectations can still relatively easily be influenced by new experiences [10, 11]. This suggests that middle childhood might be a sensitive period for interventions targeting trust in parental support. As a proof-of-principle test of the hypothesis that OT and CBM training can contribute to trust development in middle childhood, the current study aims to explore the effects of the (combination of) interventions within a sample of normally developing middle childhood participants. If the interventions prove effective in increasing trust, they could be a valuable addition to clinical interventions targeting trust in parental support.

Existing early childhood attachment-related interventions mostly focus on enhancing parental sensitivity and parents’ mental representations of the attachment relationships with their own parents [9, 12]. However, theory and research point to the importance of children’s own cognitive attachment representations and related information processing biases for repairing breaches in trust [1, 13]. More specifically, it has been shown that information regarding the parent is attended, interpreted and remembered in congruence with children’s attachment-related expectations [14–16]. As a result, interventions focusing solely on improving parenting skills might be less effective when children have insecure expectations. That is, if parents alter their behavior to be more sensitive and responsive, children who lack trust might not be able to adequately encode their parents’ retrained behavior because their insecure expectations were not altered. In support of such claims, research indicates that effect sizes of parenting training are small to moderate [17]. Moreover, effect sizes decrease when children’s age increases [18, 19], suggesting that parenting training has less impact when attachment-related information processing becomes increasingly insecure in response to prolonged exposure to insensitive and unresponsive parenting. Therefore, attachment processing biases could be an important additional target for interventions that aim to build or restore trust between parent and child.

A promising paradigm to change the way that information is processed and encoded is that of CBM [20]. CBM procedures aim to modify information processing styles by systematic practice of a target processing bias [21]. For anxiety disorders, among others, the CBM method has been shown effective in changing selective information processing biases and reducing disorder symptoms [22]. Recently, a CBM training paradigm was developed for children aged 8–12 years with the purpose to modify children’s interpretation of attachment-related information [23]. De Winter et al. [23] tested the effectiveness of this attachment-related CBM training. In their study, half of the participants received a CBM training, during which children were trained to securely interpret story scenarios that were ambiguous as to whether mother provided support. The other half of the participants received a neutral placebo training in which resolutions of ambiguity were not related to maternal behavior. Results showed that the CBM procedure was effective. That is, children in the CBM training condition showed an increase in secure interpretation bias and a decrease in insecure interpretation bias as compared to children in the neutral training condition. Importantly, trust was causally affected by attachment-related interpretation bias, as indicated by a significant increase in trust in the CBM training condition [23]. These results suggest that the CBM paradigm has therapeutic potential in the context of insecure attachment and lack of trust, as it could help build or restore children’s trust in their parents’ availability. An important next step towards the implementation of CBM as an intervention procedure is to identify the parameters that can increase the effectiveness of CBM [22].

A possible candidate for augmentation of the effects of CBM is the neuropeptide OT. Endogenous OT is synthesized in the hypothalamus, while exogenous OT can be noninvasively administrated in humans by means of a nasal spray. Functional brain imaging studies have shown that intranasal OT can modulate activity in, and connectivity between, brain regions involved in social cognition [24–26]. Overall, studies have shown that OT can help form, restore or maintain social relationships. As a result, OT has been proposed as a possible (addition to) treatment of disorders characterized by socioemotional difficulties [27]. For the current study, we propose that OT could enhance CBM effects on two levels, in accordance with the two-level model of OT effects on social behavior and social cognition as proposed by Quintana et al. [28]. First, on a behavioral level, OT has been shown to increase trust in social interaction [29] and improve attachment security [30, 31]. Therefore, OT could independently have an effect on the trust- and attachment-related outcome measures targeted by the current CBM training. Second, research suggests that OT can increase the salience and reinforcing value of socially relevant stimuli [32, 33] and enhance the processing of social information (see [34], and also [35] for neural evidence in rodents). Since the scenarios in the CBM training all describe social situations in which children might need their mother’s support, we hypothesize that OT might improve the processing of these scenarios. This way, OT could increase learning during CBM training, resulting in an additive effect of OT and CBM training on trust and trust-related outcome measures in the current study.

In pediatric populations, the majority of the limited number of OT studies has targeted children diagnosed with autism spectrum disorder (ASD) and these studies yielded mixed results [24, 36–39]. However, the scope of OT as potential therapeutic intervention reaches beyond the social problems associated with ASD [40]. Moreover, most OT studies in children were limited in sample size and sample selection. Therefore, researchers generally agree that OT’s precise role and (additive) value need further investigation [40, 41].

## The present study: aims and hypotheses

In the current study, participants administer a nasal spray that either contains OT or a placebo (PL). Additionally, they receive either a CBM training aimed at positively changing trust and related interpretation bias or a neutral training that is conjectured to have no trust-related effects. The aim of the present study is to test the main and interaction effects of CBM training and OT on three levels of trust-related outcome measures in middle childhood: trust-related interpretation bias; self-reported trust; and children’s trust-related behavior towards mother.

At the first level, we investigate the effects of CBM training, OT and both interventions combined on secure attachment interpretation bias and insecure attachment interpretation bias, as compared to no intervention (i.e., neutral training and PL). Based on the study by De Winter et al. [23], we expect that participants who receive CBM training show an increase in secure interpretation bias and a decrease in insecure interpretation bias as compared to children who receive no intervention. Since this is the first study examining the effects of OT on attachment interpretation bias, it is an empirical question as to what the effect of OT on interpretation bias is. When OT has a positive effect on interpretation bias, this should be reflected in increased secure interpretation bias and decreased insecure interpretation bias for children receiving OT as compared to no intervention. Given the proposed role of OT in improving the salience and processing of social stimuli [32, 34], intranasal administration of OT could enhance processing of the social stimuli presented in the CBM training and thereby increase its effectiveness. When joint exposure to CBM training and OT is beneficial, children in the combined intervention condition should show increased secure interpretation bias and decreased insecure interpretation bias as compared to participants who receive only one of the interventions.

At the second level, the effects of CBM training, OT, and both interventions combined on change in self-reported trust from pre-intervention to post-intervention are assessed. It is expected that participants who receive any intervention (CBM training, OT, or the two combined) will show an increase in trust as compared to participants receiving no intervention [23, 29]. If joint exposure to the two interventions is beneficial, this should be reflected in a stronger increase in trust for participants who receive the interventions combined as compared to participants who receive only one of the interventions.

At the third level, intervention-related changes in behavior of children towards mother and mother-child interactional behavior during a distressing task are observed. The main effects of the two interventions as well as the interaction effect of the interventions are assessed. To our knowledge this is the first study examining the effects of CBM training and OT on behavior during mother-child interaction. Nevertheless, previous studies have linked more trust to more positive behavior of child towards mother [42] and to a more coherent mother-child interactional pattern [43]. Therefore, if the interventions affect trust and these effects extend to the behavioral level, children who receive any intervention (CBM training, OT, or the two combined) should show an increase in positive approach towards mother as compared to children who receive no intervention. Similarly, mother-child dyads in the intervention groups (CBM training, OT, or the two combined) should show increased interactional coherence compared to mother-child dyads in which children receive neutral training and PL as a control condition.

Although the main goal of the current study is to test the effects of CBM training and OT on the abovementioned trust-related outcome measures, we plan to take the opportunity to carry out some additional explorative analyses. More specifically, we plan to test whether differences in participant characteristics moderate the effects of the interventions in the present study. This is especially relevant for the OT intervention, since accumulating evidence suggests that the effects of intranasally administered OT can be influenced by a variety of factors [44], including among others caregiving experiences [26, 45], social functioning [46], and (early) adverse events [47]. Since the present study is the first to test the effects of OT, as individual intervention and combined with CBM, on trust-related outcomes in middle childhood, it is an opportunity to explore the effects of participant characteristics to inform future research about possible factors to take into account when administering OT as an (additive) intervention. Importantly, we also closely monitor the side-effects of a single administration of OT in middle childhood to provide further information on the safety of OT administration in this age group.

## Methods/design

### Study design

The current study is a double-blind randomized controlled trial (RCT). The trial has a 2 × 2 factorial design, which combines the active and placebo interventions resulting in four equally sized intervention groups: (1) CBM training and OT (both interventions), (2) CBM training and PL (cognitive intervention only), (3) neutral training and OT (pharmacological intervention only) and (4) neutral training and PL (no intervention). The current study design allows assessment of the effects of each intervention separately and of both interventions combined.

The pharmacy of the University Hospital of Heidelberg, Germany, carried out the randomization of the nasal sprays using RITA software version 1.30 [48]. They stratified randomization by training condition and used permuted blocks within strata [49]. The bottles containing OT nasal spray and PL look identical and the pharmacy of the University Hospital of Heidelberg numbered all nasal sprays. For each nasal spray number, a sealed envelope contains a piece of paper that connects the spray number to one of the interventions. Additionally, all envelopes contain a black piece of paper to prevent visibility of intervention allocation without opening the envelope. We assign the numbered nasal sprays to consecutive participants in sequential order, with training condition alternated between participants. Since both interventions are placebo-controlled, participants are blind to the intervention condition that they are in. Experimenters are aware of the training condition, but blind to the nasal spray condition. To conceal intervention allocation and maintain blinding, we do not open the envelopes until after data collection is finished. Only in case of severe adverse events, it is possible to open a sealed emergency unblinding envelope to unblind the medication for a given participant. We assess children’s awareness of the experimental condition (i.e., both the training and the nasal spray condition) that they are in at the end of the procedure. Furthermore, we assess experimenter’s and mother’s awareness of the nasal spray condition at the end of the procedure and 24 h after the procedure, respectively. The mother-child dyad is given a participant number that we use on all data documents. A separate password-protected document links participant names to participant numbers. In case of severe adverse events after nasal spray administration, the procedure can be discontinued. Moreover, mother and child can withdraw from the study at any time without giving a reason.

The trial takes place at the Parenting and Special Education Research Unit, KU Leuven, Belgium. The Medical Ethics Committee UZ KU Leuven/Research (S57012) approved the study design in accordance with the Declaration of Helsinki. We submit any protocol amendments and progress reports to the Ethics Committee as required, as well as an end-of-study declaration. We registered the trial in the database of ClinicalTrials.gov (NCT02737254). The Belgian Federal Agency for Medicines and Health Products also approved the trial. See Additional file 1 and Table 1 for a checklist and an overview of study procedures in line with Standard Protocol Items: Recommendations for Interventional Trials (SPIRIT) [50] and Additional file 2 for a description of all items from the World Health Organization Trial Registration Data Set.

**Table 1:** Schedule of enrollment, interventions and assessments in line with Standard Protocol Items: Recommendations for Interventional Trials (SPIRIT)

	Study period				
	Enrollment	Allocation	Post-allocation		
Timepoint	Before intervention (baseline)	0	During intervention	Immediately after intervention	24 h after intervention
Enrollment:					
Eligibility screen	X				
Informed consent	X				
Allocation		X			
Interventions:					
CBM training + OT			X		
CBM training + PL			X		
Neutral training + OT			X		
Neutral training + PL			X		
Assessments:					
Interpretation speed of secure and insecure scenario resolutions			X		
Secure interpretation bias	X			X	
Insecure interpretation bias	X			X	
Trust (PIML)	X			X	
Puzzle task	X			X	
Mood	X		X	X	
OT side-effects				X	X
Mother-reported questionnaires:					
Demographic questionnaire			X		
Child temperament (EATQ-R)			X		
Child behavioral and emotional problems (CBCL)			X		
Parenting questionnaires (VSOG, subscales LAPPS, POPS, PCS)			X		
Pubertal development (PDS)			X		
Symptoms of sino-nasal problems (Snot-20)			X		

*CBCL* Child Behavior Checklist, *EATQ-R* Early Adolescent Temperament Questionnaire-Revised, *LAPPS* Louvain Adolescent Perceived Parenting Scale, *OT* oxytocin, *PCS* Psychological Control Scale, *PDS* Pubertal Developmental Scale, *PIML* People In My Life, *PL* placebo, *POPS* Perceptions of Parents Scale, 20-*Snot-20* 20-item Sino-Nasal Outcome Test, *VSOG* Verkorte Schaal Ouderlijk Gedrag

### Participants

We plan to recruit 100 children (boys and girls) and their mothers. The inclusion and exclusion criteria for the children are listed below:

Inclusion criteria:

- Age 8–12 years
- Capable of comprehending and reading the Dutch language

Exclusion criteria:

- Known OT allergy
- Currently using medication
- Kidney or cardiac condition

### Procedure

We will recruit children via the distribution of flyers at local schools, youth clubs and events, the local university and hospital. After stating interest to participate, mothers receive information about the study in general and about OT specifically via email. Subsequently, the researchers contact mothers by phone and explain the procedure verbally, check inclusion and exclusion criteria and address any questions or concerns. When eligible, we invite mother and child to participate and schedule a testing appointment. We ask mothers to make sure that the child abstains from caffeine for at least 2 h before study onset.

**Fig. 1:**
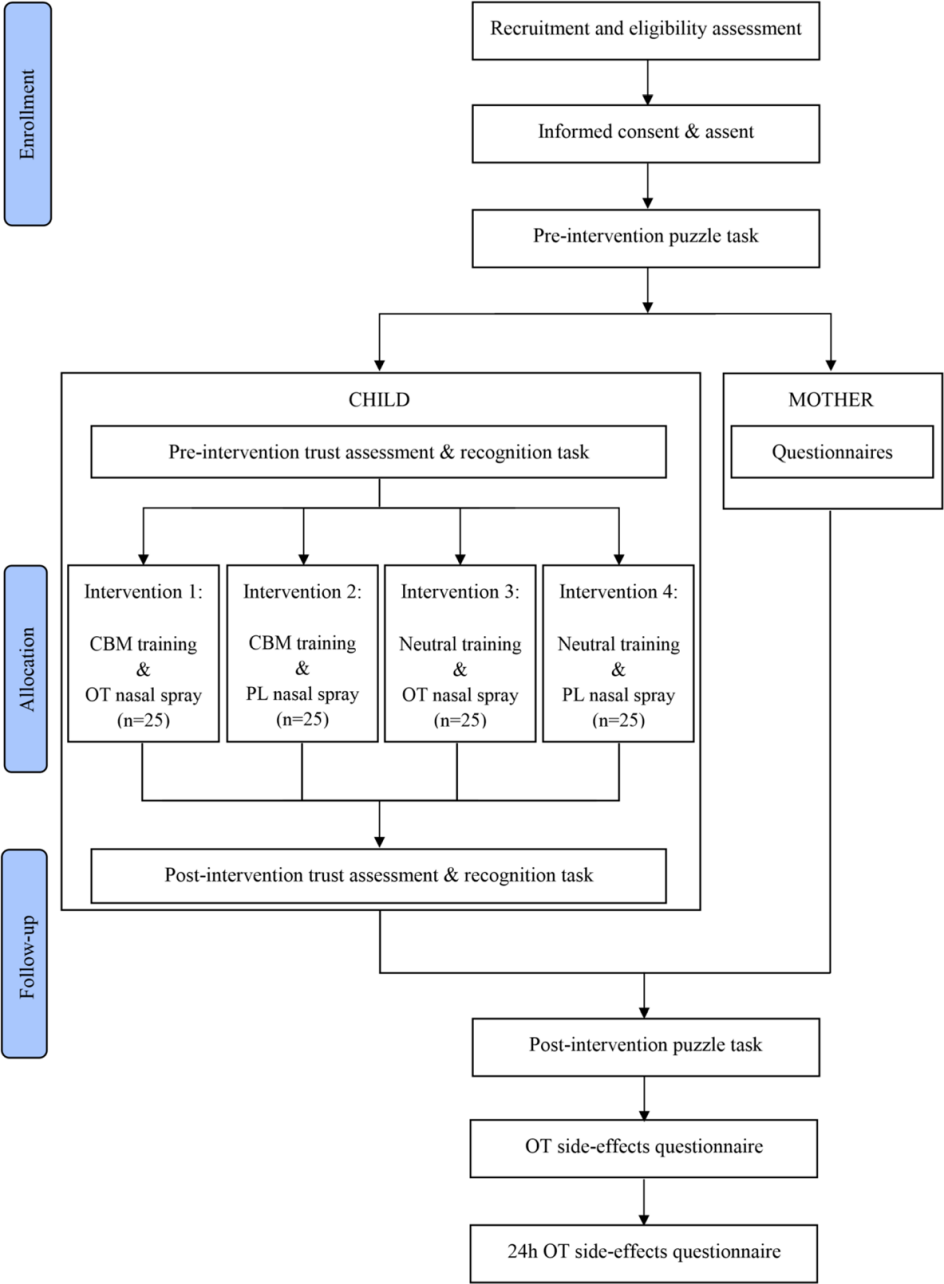
Participant flowchart

Upon arrival at the laboratory, mother and child complete a written informed consent (see Additional file 3 for the Informed Consent Form) and assent respectively. The child’s procedure consists of (1) a pre-intervention puzzle task, (2) a pre-intervention trust questionnaire, (3) a pre-intervention recognition task, (4) nasal spray administration, (5) a 35-min break, (6) training, (7) a post-intervention recognition task, (8) a post-intervention trust questionnaire, (9) a post-intervention puzzle task and (10) a side-effects questionnaire. The mother is present for the pre-intervention puzzle task, after which she retreats to a different room to fill out a questionnaire booklet. She only returns when it is time to start with the post-intervention puzzle task. The entire procedure lasts approximately 120 to 150 min. At the end of the procedure, participants receive two cinema tickets in gratitude for their participation. See Fig. 1 for a flowchart of the child’s and mother’s procedure.

### Interventions

#### OT intervention

The pharmacy of the University Hospital of Heidelberg, Germany prepares the study medication. Half of the participants receives OT (Syntocinon®, 40 international units (IU)/ml), the other half receives PL (0.9% sodium chloride solution). The bottles containing OT and PL are identical: 10-ml brown glass bottles, hydrolytic class type III, with DIN ND 18 screw thread. The nasal pump has a volume of 0.05 ml per puff. We weigh children on a digital personal scale and base the amount of nasal spray administered on the child’s weight. The conventional dose in adult studies is 24 IU OT, although a study comparing different doses in adults has shown that salivary OT levels were equally elevated after intranasal administration of 24 IU OT and of 16 IU OT [51]. Studies involving OT nasal spray in children (under 12 years) used doses varying between 8 IU and 24 IU. These studies based the amount of nasal spray administered on age as a proxy for weight [24], on weight [36], or gave all children the same (stepwise raised) amount of OT [39]. These studies either found that the results did not depend on OT dosage [36, 39] or did not report on dosage effects [24]. As it is most common to base drug doses in pediatric populations on weight [52], we used the same weight-based dosing strategy as in the study by Dadds et al. [36]: children weighing less than 40 kg receive 0.3 ml nasal spray (i.e., 12 IU OT for the OT condition); children weighing more than 40 kg receive 0.6 ml (i.e., 24 IU OT for the OT condition). Single administration of OT in dosage amounts up to 40 IU has not resulted in side-effects or adverse outcomes [53].

We based the nasal spray administration procedure on recommendations by Guastella et al. [54]. Before use, the experimenter primes the spray by pumping approximately seven to eight times until a fine mist is visibly released. After priming, the experimenter weighs the bottle on an apothecary scale (Kern EMB 200-2). Experimenters ask the children to keep their head upright or slightly tilted backwards during administration. Experimenters explain to the children that we will alternate administration between nostrils and that they have to keep the other nostril closed with one finger and inhale lightly two or three times upon nasal spray delivery. Child and experimenter practice this administration procedure together before actual administration. Either the experimenter, or the child itself under experimenter supervision, administers the spray. Experimenters make sure that the top of the nasal spray is inserted into the nose about 1 cm and that the bottle is held at a slight angle such that the pipe is well immersed in the fluid at all times. After 6 (for children under 40 kg) or 12 (for children over 40 kg) pumps, the experimenter weighs the spray again on the scale. If necessary, we administer extra pumps until at least 0.26 ml or 0.56 ml has been released from the bottle. Experimenters register the amount of nasal spray administered.

Subsequently, children wait for 35 min, during which they can choose to draw, read a comic or watch a video. During the break, experimenters make sure that children remain calm (e.g., do not watch exciting videos), do not eat or drink except for water, and are not in contact with their mother.

#### CBM intervention

After the break, children start a computerized training that either aims to positively modify their interpretation of maternal support-related behavior and subsequent trust in maternal support (CBM training condition) or aims to have no effect (neutral training condition). The same CBM training was effective in modifying attachment interpretation bias in the study by De Winter et al. [23]. During training (both conditions) children read, at their own pace, 42 short scenarios on a laptop. The scenarios describe situations in which middle childhood children might need maternal support [55]. All scenarios consist of three lines of text and in the last line a word is missing, rendering the outcome of the scenario ambiguous. On the next screen, the missing word is presented as a word fragment (i.e., one letter in the word is missing). The child’s task is to figure out what the word should be and hence which letter is missing. Children press the space button as soon as they know the missing letter. Subsequently, they press the missing letter on the keyboard. To make sure that children actively read the scenarios, they have to answer a yes/no comprehension question after each scenario, after which they receive feedback on their answer. The training consists of six blocks of seven scenarios each and children can take a short break in between blocks.

Every block consists of two probe scenarios that are the same for the two conditions (see “Outcome measures” below) and five scenarios that are different for the two conditions. In the CBM training condition each block contains five training scenarios that are aimed at increasing secure interpretation bias. As a result of the missing word in the last sentence it is unclear whether mother does or does not provide support. In these training scenarios the resolving word fragment is always secure, which trains the child to interpret ambiguous maternal behavior in a secure way. In the neutral training condition, each block contains five neutral scenarios. The first two sentences in these scenarios are similar to the training scenarios in the CBM condition. The last sentence, however, is unrelated to maternal support. Therefore, resolving the word fragment does not influence the child’s interpretation of mother’s behavior. For learning effects to occur, it is important to provide enough repetition for participants to be able to learn to interpret the ambiguous scenarios in a positive way, while at the same time not to overload participants in order to keep them attentive and motivated. The current distribution of 42 scenario’s over six blocks was both feasible and effective in modifying attachment interpretation bias and trust in the study by De Winter et al. [23].

### Measures

#### Outcome measures

An overview of outcome measures is presented in Table 2.

**Table 2:** Outcome measures

Domain	Specific measurement	Metric	Method of aggregation	Time point		
				Baseline	Training	Post-intervention
Interpretation bias	Interpretation speed of secure and insecure scenario resolutions	Difference between secure and insecure resolutions	Mean reaction time to secure and insecure probes		x	
	Secure interpretation bias	Change from pre-intervention to post-intervention recognition task	Mean rating of secure interpretations	x		x
	Insecure interpretation bias	Change from pre-intervention to post-intervention recognition task	Mean rating of insecure interpretations	x		x
Trust	PIML Trust subscale	Change from pre-intervention to post-intervention	Mean rating of trust items	x		x
Child’s attachment-related behavior	Observation of the quality of the approach of child towards mother during a puzzle task with the MCAM scoring scales	Change from pre-intervention to post-intervention	Frequency of child behaviors	x		x
Mother-child interactional coherence	Observation of positive parent behavior and positive child behavior (with the MCAM scales) during a puzzle task	Change from pre-intervention to post-intervention	Jaccard Index indicating the occurrence of parent behavior at time point t − 1 and child behavior at time point t and vice versa (i.e., indicating the occurrence of child behavior at time point t − 1 and parent behavior at time point t)	x		x

*PIML* People In My Life; *MCAM* Middle Childhood Attachment Micro-observation system

##### Interpretation bias

We assess interpretation bias with two different measures: interpretation speed of secure and insecure scenarios, and spontaneous interpretation of new attachment-related information.

##### Interpretation speed of secure and insecure resolutions

We assess interpretation speed of secure and insecure resolutions of ambiguous maternal behavior during training. As aforementioned, in both conditions, each block contains two probe scenarios: one secure and one insecure (12 probes in total). The format of the probe scenarios is the same as that of the training scenarios. For the secure probes, the resolution of the scenario is always secure (i.e., mother provides support). For the insecure probes, the resolution of the scenario is always insecure (i.e., mother does not provide support). We randomized presentation of the probe and training trials within each block, with the final order kept identical for all participants. We test differences between intervention groups in interpretation speed of secure versus insecure scenarios using mean reaction times to secure and insecure resolving word fragments separately. Faster mean reaction times to secure probes and slower mean reaction times to insecure probes indicate a more secure interpretation bias.

##### Change in secure interpretation and insecure interpretation of ambiguous maternal behavior

Before and after intervention we measure children’s spontaneous interpretation of ambiguous maternal behavior by means of a recognition task [23, 56]. During this task children read seven 3-line scenarios and, as in the training, a word is missing in the last line. The next screen shows the missing word as a word fragment and children have to press the space button as quickly as possible when they can complete this fragment. Subsequently, they press the missing letter on the keyboard. After resolving the word fragment, however, it remains ambiguous whether or not mother provided support. To make sure that children actively read the scenarios, they have to answer a yes/no comprehension question after each scenario followed by feedback on their answer. Once children have read all seven scenarios, we measure their interpretation bias. Specifically, for each scenario, two events are described: one secure event (i.e., describing supportive maternal behavior) and one insecure event (i.e., describing unsupportive maternal behavior). For each event, children have to indicate to what extent they think the described event occurred in the scenario on a 4-point Likert scale ranging from 1 (completely untrue) to 4 (completely true). However, none of the events actually occurred in the scenarios. Therefore, the scores measure children’s spontaneous interpretation of ambiguous maternal behavior. Change in secure and insecure interpretations is examined separately. An increase in scores for the secure events means a more secure interpretation bias. Likewise, an increased score for the insecure events indicates a more insecure interpretation bias.

##### Change in trust

We measure trust in mother with the Trust subscale of the People In My Life Questionnaire (PIML) [57]. This subscale contains 10 items concerning experiences with mother as a trustworthy source for support (e.g., “I can count on my mother to help me when I have a problem”). Children have to indicate how true the item is for them on a Likert scale ranging from 1 (almost never true) to 4 (almost always true). We administer the subscale before intervention to determine baseline trust in maternal support. Shortly after intervention we readministered the scale to allow assessment of change in trust. The PIML is a valid self-reported measure for middle childhood [14] and has been used in previous research to assess change in trust in maternal support [23].

##### Change in observed behavior during a distressing task

We assess changes in behavior of children towards mother and in mother-child interactional behavior by means of videotaped observation of mother-child interaction during a distressing puzzle task. Researchers have used this puzzle task, taken from Rush Hour Traffic Jam Puzzle®, before to observe attachment behavior in a distressing situation in middle childhood [58, 59]. In this task, a red car’s path out of a traffic jam is blocked by other cars and trucks. The child has to move the other cars to free the path for the red car. After the experimenter explains the rules, children first solve a simple practice puzzle. Subsequently the child receives an unsolvable puzzle with the instruction that their mother is allowed to help. The experimenter tells children that the puzzle is not very difficult, that most children their age can solve the puzzle in less than 5 min, and that, therefore, they are also given 5 min to solve the puzzle. A laptop screen shows a stopwatch so that the child can see how much time is left. The combination of trying to solve an unsolvable puzzle under time pressure and social comparison with peers effectively induced stress in previous research [59]. After starting the stopwatch the experimenter leaves the room. Three minutes later the experimenter returns and aborts the attempt to solve the puzzle with the excuse that, on account of the timing of the procedure, they have to proceed with the rest of the tasks. The experimenter then says that the mother-child dyad will be given another chance to solve the puzzle when there is time left at the end of the procedure. The attempt to solve the puzzle is terminated after 3 min instead of 5 min so children do not specifically fail on the first attempt, but are merely given too little time to solve it. This with the aim to increase comparability between the pre-intervention and post-intervention puzzle tasks.

We administer the same puzzle task post-intervention. Participants receive the same instructions as pre-intervention, after which the experimenter starts the stopwatch and leaves the room. Three minutes later the experimenter enters again and stops the attempt to solve the puzzle by asking the mother-child dyad whether they already succeeded in solving the puzzle. As a manipulation check the experimenter asks – first the child, then the mother – if they know how it is possible that they were not able to solve the puzzle.

We videotape the pre-intervention and post-intervention puzzle task and code mother and child’s verbal and nonverbal behavior by means of the Middle Childhood Attachment Micro-observation system (MCAM) [42]. The MCAM was based on the emotional attachment scales (EAS middle childhood version) [60] and the Strange Situation Procedure (SSP) [3], and researchers have used it before to register mother-child interactions during a similar puzzle task [42, 43]. The MCAM distinguishes six global child behavior categories: responsiveness; involving the parent (i.e., proximity-seeking); clinging and controlling behavior; closeness and interaction avoidant behavior; closeness and interaction opposing behavior; and puzzle behavior (together versus alone). In the present study, we use partial-interval recording to code the occurrence of behaviors in all MCAM categories every 2 s [61]. We assess frequency of these behaviors for the pre-intervention and post-intervention puzzle task to evaluate change in trust-related behavior towards mother due to the interventions.

To assess change in mother-child interactional coherence, we also code mother’s behavior using partial-interval recording. The MCAM distinguishes five global parent behavior categories: parental sensitivity; parental structuring; noncontingent response; parental hostility; and puzzle behavior (together versus alone). Using the Jaccard Index [62], we assess the extent to which positive parent behavior (as coded in the categories parental structuring and parental sensitivity) at time point t − 1 is predictive of positive child behavior (as coded in the categories responsiveness and involving the parent) at time point t. Additionally, we explore whether positive child behavior at time point t − 1 is predictive of positive parent behavior at time point t. Coders are blind to the intervention condition (both training and nasal spray condition) that the mother-child dyads are in.

### Other measures

#### Oxytocin side-effects

We explore any side-effects of the OT intervention with parent and child questionnaires. All children are asked about the most common reported side-effects which include dizziness, drowsiness, dry mouth, irritated or runny nose, stomach- or headache, anxiety, feeling uplifted and feeling relaxed [53], and any other noted changes immediately after the procedure (face-to-face). After 24 h, mothers answer the same questions via phone. Experimenters register the onset, duration and severity of any reported side-effects.

#### Mood

Before and after intervention, children rate their mood on two Visual Analogue Scales (VAS; 100 mm). One scale assesses to which extent children are in a happy mood, the other measures to which extent they are in a sad mood. Additionally, children fill out these mood scales immediately before training (i.e., 35 min after nasal spray administration). We can use the mood measurements to assess effects of the interventions on mood, to control for possible mood effects on the primary and secondary outcome measures.

#### Mother-reported questionnaires

We administer the following mother-reported questionnaires to allow for exploratory moderation analyses of the intervention effects.

A demographic questionnaire collects participant characteristics including among others gender, date of birth, living situation, nationality, birth weight, length of pregnancy and complications during pregnancy, attachment figures during first years of life, parental separation, parental level of education and parental socioeconomic status.

The Early Adolescent Temperament Questionnaire-Revised (EATQ-R) assesses child temperament [63]. The EATQ-R consists of 62 items (e.g., “My child finds it easy to really concentrate on a problem”) that mothers rate on a 5-point Likert scale. The psychometric properties of the EATQ-R are sufficient [64].

We assess children’s behavioral and emotional problems with the Child Behavior Checklist (CBCL) [65, 66]. The CBCL consists of 113 items (e.g., “Fights a lot”) for which mother rates on a 3-point scale to what extent they are true for their child in the last 6 months. The reliability and validity of the CBCL is well established [67].

Two questionnaires, comprising in total 47 items, assess mother’s parenting style. The short version of the Ghent Parental Behavior Questionnaire (“Verkorte Schaal Ouderlijk Gedrag” (VSOG); 25 items [68]) taps into mother’s parenting behavior. A second questionnaire consists of three subscales: the Responsiveness subscale of the Louvain Adolescent Perceived Parenting Scale (LAPPS; seven items [69]), a Dutch version of the Autonomy Support subscale of the Perceptions of Parents Scale (POPS; seven items [70]) and a Dutch version of the Psychological Control Scale (PCS; eight items [71]). Mothers rate the items on a 5-point Likert scale. Previous research has shown that the (Dutch versions of these) questionnaires and subscales are reliable [68, 72–74].

We assess children’s pubertal status with the parent-reported Pubertal Developmental Scale (PDS) [75]. Mothers rate five items about their child’s physical development (separately for boys, e.g., beard growth, and girls, e.g., breast development) on a 5-point scale. Past research found significant associations between the parent-reported PDS and the self-reported PDS [76]. The PDS has good reliability and validity [75, 77].

We evaluate symptoms of sino-nasal problems in the last 2 weeks with a parent-reported brief version of the 20-item Sino-Nasal Outcome Test (Snot-20) [78]. Mothers indicate how much their children were affected by symptoms of sino-nasal problem (10 items; e.g., “need to blow nose”) in the past 2 weeks on a 5-point scale.

#### Data management

Data is either saved automatically in E-prime data files (for the interpretation bias measures) or the experimenters enter data manually into SPSS data files (for all other measures). We assure the quality of data entry by random checking of the data entered. After each day of testing we will back-up the data on the first author’s personal KU Leuven network drive which the KU Leuven IT department in turn automatically backs-up daily.

There is no data monitoring committee due to the characteristics of the current study: a nonclinical participant group, tested for a short period of time with intervention(s) that have not been reported previously to harm participants [53]. There are no interim analyses and stopping guidelines planned.

Study data is only accessible by the researchers involved in the trial. Feedback on how an individual participant results is not possible; however, we plan to communicate summarized general results to parents who are interested. Parents are informed about this option in the informed consent.

#### Data analysis

For all analyses, we consider effects for which *p* values < .05 as statistically significant. We assess baseline comparability of the groups using descriptive statistics (gender, age). We use one-way analyses of variance (ANOVAs) to assess pre-intervention differences between the four intervention conditions on the outcome measures. Data that is missing completely at random will be calculated using Expectation Maximization.

The aim of the present study is to explore the effectiveness of CBM training, OT and both interventions combined on trust in maternal support on three levels: trust-related interpretation bias; self-reported trust; and mother-child interactional behavior. To assess the main and interaction effects of the interventions on change scores on these outcome measures, we will perform a two-way ANOVA with training condition (CBM versus neutral) and nasal spray condition (OT versus PL) as between-subject variables. Only in case of the measure of interpretation speed of secure and insecure scenario resolutions, we add the within-subject factor probe valence (secure versus insecure). Additionally, in order to check whether the dose of intranasal OT that is administered affects intervention effects on change scores on the outcome measures, we plan to run a two-way ANOVA in the OT intervention condition with training condition (CBM versus neutral) and OT dose (12 IU versus 24 IU) as between-subject variables and children’s weight as covariate.

#### Sample size

The current study is powered to test the main hypotheses; that is, to identify a difference (moderate effect size, *f* = 0.30) in outcome measures between the four treatment conditions. Since this was the first study to combine CBM and OT interventions to investigate their main and interaction effects on trust-related outcome measures, there were no effect sizes from comparable studies that we could use to calculate the sample size needed. However, relevant prior studies point to the fact that a moderate effect size might be expected. Specifically, previous studies using the same CBM training obtained effect sizes ranging from *f* = 0.29 to *f* = 0.48 for trust and trust-related outcome measures [23, 79]. Additionally, a meta-analysis of effects of OT administration to in-group trust showed an effect size *f* = 0.24, with an effect size *f* = 0.32 for studies with a between-subject design such as the current study [33].

We used G*power 3.1 [80] to computed the required sample size for an ANOVA test for main effects and interactions. Input parameters were: a moderate effect size, *f* = 0.30; alpha = .05, two-tailed; power = 0.80; numerator *df* = 1; number of groups = 4. G*power revealed that a sample size of 90 participants is required to have a power of 0.80 to identify a moderate effect between the four treatment conditions (critical *F* = 3.95). We plan to recruit 100 participants in total (25 in each intervention condition), yielding an actual power of 0.84 (critical *F* = 3.94).

## Discussion

Given the association between lack of trust in the parent and a wide range of psychopathological problems across the lifespan, there is need for interventions that can help build trust in parent-child dyads. The present article describes the protocol for a study that aims to test the individual and combined effects of two interventions – CBM training and intranasal OT – on children’s trust-related information interpretation bias, trust in maternal support, and observed mother-child interactional behavior. To this purpose, children are administered a nasal spray that contains either OT or PL. In addition, they receive either a CBM training aimed at positively changing trust and related interpretation bias or a neutral training that aims to have no trust-related effects. The different outcome measures are administered pre- and post-intervention to allow assessment of change due to the interventions.

The present study is the first study to combine intranasal OT administration with a CBM training procedure. Both individually and combined, the two interventions could exert effects on trust. The current study’s factorial design allows for assessment of these combined and individual effects of CBM training and OT. Other strengths are the double-blind and randomized design of the trial, reducing the risk of bias. Moreover, to our knowledge, the current trial is the first OT study in general-population, middle-childhood children with sufficient power to detect moderate OT effects.

In line with this, an important additional aim of the present study is to increase our knowledge of adverse events or side-effects associated with OT administration in pediatric populations. To date, limited data is available on the side-effects of OT in children, due to a small number of studies performed with limited sample sizes [40]. In the present study, the side-effects of OT are monitored in a relatively large sample of middle-childhood children, both at the end of the procedure and 24 h after intervention. The current trial can thus provide more insight into any side-effects associated with single-dose OT in middle-childhood children, which is an important step towards the use of OT as an intervention in pediatric populations.

Finally, the study provides the opportunity to explore potential moderators of the effects of the interventions. It must be noted here that the sample size of the current study was budgeted to have adequate power to test the main hypotheses. However, accumulating evidence suggests differential effects of intranasally administered OT depending on a variety of participant characteristics [26, 44–47]. Because this will be the first study with OT in such a large, normal-population, middle-childhood sample, we considered it a missed opportunity not to include potential moderators of OT effects and to perform preliminary exploratory analyses. This might be relevant for future research as it could point to relevant factors to take into account in OT research in this sample. However, lack of power will make it hard to draw conclusions from moderation analyses and, therefore, research in larger samples will be needed to further examine possible moderators. Additionally, although we assess a range of possible moderators in the current study, there are other factors that we did not examine that could affect the working of the OT nasal spray. An interesting question for future research might, for instance, be whether (exclusive) breastfeeding and mode of birth affect children’s responsiveness to intranasal OT even years later [44, 81].

A limitation regarding the current study design is that effects of the interventions are assessed immediately after intervention. As a result, prolonged long-term effects of the interventions remain unknown. Similarly, since the current study consists of a single session, we cannot assess the impact of multiple sessions of the intervention(s). Without doubt, longer-term effects of OT administration and CBM are important to assess when considering them as clinical interventions to improve or rebuild trust. However, since previous research using the same CBM procedure yielded immediate effects on trust c, the current study was set up to test (1) whether OT can enhance these CBM effects and (2) whether OT as individual intervention has an immediate effect on trust. As the present study is the first to combine these two interventions, it is an important first step to assess the short-term effects and feasibility of a single intervention session. Nonetheless, if the (combined) interventions were to prove effective in the current study, future research will be crucial to explore the long-term effects of the interventions as well as the effect of multiple intervention sessions. Additionally, the effects of the interventions are tested in a normal population sample and, therefore, the effectiveness of the interventions in clinical groups cannot be ascertained from the current study. If the current study were to provide favorable results following the CBM and OT interventions, another important next step is to assess effects of the interventions in clinical samples.

In all, the current paper describes the protocol for a RCT investigating the individual and combined effects of CBM training and OT on trust in mother. Additionally, the current RCT tests the safety of OT administration in 8–12-year-old children. As OT studies in pediatric populations have yielded mixed results, with the current comprehensive protocol description we aim to expedite the clarity and reproducibility of OT research findings. If effective, CBM training and OT could be easily applicable and nonintrusive additions to interventions that target trust in the context of the parent-child relationship.

### Trial status

Mother-child dyads began to enter the trial in March 2016. Recruitment is still open.

## Additional files

Additional file 1: SPIRIT 2013 Checklist: recommended items to address in a clinical trial protocol and related documents. (DOC 121 kb)

Additional file 2: Items from the WHO Trial Registration Data Set. (DOCX 15 kb)

Additional file 3: Informed Consent Form-mother (Dutch original and English translation). (DOCX 20 kb)

## Abbreviations

ASD: Autism spectrum disorder
CBCL: Child Behavior Checklist
CBM: Cognitive Bias Modification
EAS: Emotional attachment scales
EATQ-R: Early Adolescent Temperament Questionnaire-Revised
IU: International unit
LAPPS: Louvain Adolescent Perceived Parenting Scale
MCAM: Middle Childhood Attachment Micro-observation system
OT: Oxytocin
PCS: Psychological Control Scale
PDS: Pubertal Developmental Scale
PIML: People In My Life Questionnaire
PL: Placebo
POPS: Perceptions of Parents Scale
RCT: Randomized controlled trial
Snot-20: 20-item Sino-Nasal Outcome Test
SSP: Strange Situation Procedure
VAS: Visual Analogue Scale
VSOG: Verkorte Schaal Ouderlijk Gedrag
