# Supplementary material for: The effects of Cognitive Bias Modification training and oxytocin administration on trust in maternal support: study protocol for a randomized controlled trial
Source: Trials. 2017 Jul 14;18:326. doi: 10.1186/s13063-017-2077-2 (PMC5513044; doi:10.1186/s13063-017-2077-2)
Supplement: Supplementary file 2 — Items from the WHO Trial Registration Data Set. (DOCX 15 kb) [file 13063_2017_2077_MOESM2_ESM.docx]

**Items from the WHO Trial Registration Data Set**

| Primary registry and trial identifying number | ClinicalTrials.gov NCT02737254 |
| --- | --- |
| Date of registration in primary registry | March 23 2016 |
| Secondary identifying numbers | S57012 (KU Leuven) |
| Source(s) of monetary or material support | Research Foundation Flanders (FWO)  Research Fund KU Leuven |
| Primary sponsor | KU Leuven, Belgium  Prof. Dr. Guy Bosmans  guy.bosmans@kuleuven.be |
| Secondary sponsor(s) |  |
| Contact for public queries | Martine Verhees, MSc., KU Leuven  Address: Leopold Vanderkelenstraat 32, 3000, Leuven Belgium  Phone: +3216321835  Email: martine.verhees@kuleuven.be |
| Contact for scientific queries | Prof. Dr. Guy Bosmans, KU Leuven  Address: Leopold Vanderkelenstraat 32, 3000, Leuven Belgium  Phone: +3216326187  Email: guy.bosmans@kuleuven.be |
| Public title | Oxytocin and attachment-related interpretation bias |
| Scientific title | The effect of oxytocin on the training of attachment-related interpretation bias in middle childhood. |
| Countries of recruitment | Belgium |
| Health condition(s) or problem(s) studied | Trust |
| Intervention(s) | Oxytocin (40 IU/mg; children <40kg receive 12IU, children >40kg receive 24IU) vs. Placebo (NaCL 0.9%)  Secure CBM training (children are trained to interpret ambiguous maternal behavior in a secure way) vs. Neutral training (children receive a training unrelated to the interpretation of maternal behavior).  Study arms:  Oxytocin and Secure CBM training (experimental)  Placebo and Secure CBM training (active comparator)  Oxytocin and Neutral training (active comparator)  Placebo and Neutral training (placebo comparator) |
| Key inclusion and exclusion criteria | Inclusion Criteria:  Children between 8 and 13 years old – mother has to be able to participate as well  Capable to comprehend and read the Dutch language  Exclusion Criteria:  Known oxytocin allergy  Currently using medication  Kidney or cardial condition |
| Study type | Interventional Allocation: randomized Intervention model: factorial assignment Masking: double blind (participant, investigator) Primary purpose: treatment Phase 4 |
| Date of first enrolment | March 2016 |
| Target sample size | 100 |
| Recruitment status | September 2016: Recruiting |
| Primary outcome(s) | Change in trust in mother as measured with the People in My Life Trust subscale [ Time Frame: Immediately before and immediately after intervention; not designated as safety issue] |
| Key secondary outcomes | Interpretation bias as measured with interpretation speed of positive vs. negative maternal behavior [ Time Frame: During training; not designated as safety issue]  Secure interpretation bias as measured with a recognition task [Time Frame: Immediately before and immediately after intervention; not designated as safety issue]  Insecure interpretation bias as measured with a recognition task [Time Frame: Immediately before and immediately after intervention; not designated as safety issue]  Change in attachment-related behavior towards mother [ Time Frame: Immediately before and immediately after intervention; not designated as safety issue] |
