# Supplementary material for: The effects of Cognitive Bias Modification training and oxytocin administration on trust in maternal support: study protocol for a randomized controlled trial
Source: Trials. 2017 Jul 14;18:326. doi: 10.1186/s13063-017-2077-2 (PMC5513044; doi:10.1186/s13063-017-2077-2)
Supplement: Supplementary file 3 — Informed Consent Form-mother (Dutch original and English translation). (DOCX 20 kb) [file 13063_2017_2077_MOESM3_ESM.docx]

**Informed consent** (English translation)

To be completed by the participants and/or is reviewed together with the researcher.

I understand what the purpose of this study is.

I know that I will participate in the following:

Mother and child try to solve a puzzle together. This task will be videotaped. The video will be watched later by the researchers.

The participating child inhales oxytocin or a placebo. The dose is dependent on the child’s weight. Children weighing less than 40 kg receive about six sprays. Children weighing more than 40 kg receive about 12 sprays.

The participating child fills out questionnaires on the parent-child relationship.

The participating child participates in a computerized training.

The participating child fills out a questionnaire about his/her experiences with oxytocin.

I fill out a demographic questionnaire.

I fill out a parenting questionnaire.

I fill out a questionnaire about my child’s problem behavior.

I fill out a questionnaire about my child’s pubertal development.

I fill out a questionnaire about my child’s temperament.

I fill out a questionnaire about any nasal problems my child might have.

I will be contacted 24 hours after the procedure by the researcher to answer some questions about my child’s experiences with the nose spray.

I know that there may be risks or inconveniences associated with my participation:

Children with a known oxytocin allergy may not participate in the study

Children currently taking medication may not participate in the study

Children with known cardiac or kidney problems may not participate in the study

Myself or others may benefit from this research as follows:

Discovering factors that can enhance the training effect can lead to a clinically relevant training

I participate in this study voluntarily.

The results of this study can be used for scientific purposes and may be published.

My name will not be published and the confidentiality of the data is guaranteed at any stage in the study.

I reserve the right to stop my participation at any time, and I know this may not lead to any harm to me.

For any questions, complaints, further follow-up, I know that after my participation, I can contact:

smec@kuleuven.be

Martine Verhees ([martine.verhees@ppw.kuleuven.be](mailto:simon.dewinter@ppw.kuleuven.be))

Prof. Kristina Casteels – pediatrician UZ Leuven ([kristina.casteels@uzleuven.be](mailto:kristina.casteels@uzleuven.be))

Research shows that there are no side effects after using oxytocin or the placebo drug. If there is any need for medical assistance during or after the study, it is always available.

The study was approved by the Medical Ethics Committee of UZ KU Leuven/ Research.

In accordance with the Belgian Law of May 7 2004 on Human Experiments, even when without mistakes, the KU Leuven is liable for all damages that may occur to the participant that is directly or indirectly related to the experiment. The commissioner of this study [KU Leuven] has an insurance covering this liability. Should you incur damage as a result of your participation in this study, this damage will therefore be reimbursed in accordance with the Belgian law of May 7 2004.

Date:

Name and signature participant: Name and signature researcher:

**Informed consent** (Dutch original)

Wordt ingevuld door de proefpersonen en/of wordt samen met de onderzoeker doorgenomen.

Ik begrijp wat het doel van dit onderzoek is.

Ik weet dat ik zal deelnemen aan volgende proeven of testen:

Ouder en kind proberen samen een puzzel op te lossen. Deze taak wordt opgenomen op video. Deze video zal later door de onderzoekers worden bekeken.

Het deelnemende kind krijgt oxytocine of een placebo toegediend. De dosering is afhankelijk van het gewicht van het kind. Kinderen die minder dan 40 kg wegen ontvangen 6 verstuivingen. Kinderen die meer dan 40 kg wegen, ontvangen 12 verstuivingen.

Het deelnemende kind vult enkele bestaande vragenlijsten in over de ouder-kind relatie

Het deelnemende kind doorloopt een computergestuurde training

Het deelnemende kind vult een vragenlijst in over zijn ervaringen met oxytocine

Ik vul een vragenlijst in over demografische gegevens

Ik vul een vragenlijst in over opvoeden

Ik vul een vragenlijst in over probleemgedrag van mijn kind

Ik vul een vragenlijst in over de puberteitsontwikkeling van mijn kind

Ik vul een vragenlijst in over het temperament van mijn kind

Ik vul een vragenlijst in over eventuele nasale problemen van mijn kind

Ik word na 24 uur gecontacteerd door de onderzoeker om enkele vragen te beantwoorden over de ervaringen van mijn kind met oxytocine

Ik weet dat er risico's of ongemakken kunnen verbonden zijn aan mijn deelname:

Kinderen met een gekende oxytocine allergie mogen niet deelnemen aan het onderzoek

Kinderen die momenteel medicatie nemen mogen niet deelnemen aan het onderzoek

Kinderen met een gekende hart- of nierproblemen mogen niet deelnemen aan het onderzoek

Ikzelf of anderen kunnen baat bij dit onderzoek vinden op volgende wijze:

Het ontdekken van factoren die het trainingseffect kunnen versterken, kan leiden tot een klinische relevante training

Ik neem uit vrije wil deel aan dit onderzoek.

De resultaten van dit onderzoek kunnen gebruikt worden voor wetenschappelijke doeleinden en mogen gepubliceerd worden.

Mijn naam wordt daarbij niet gepubliceerd en de vertrouwelijkheid van de gegevens is in elk stadium van het onderzoek gewaarborgd.

Ik behoud het recht om op elk moment mijn deelname aan het onderzoek stop te zetten en ik weet dat daaruit geen nadeel voor mij mag ontstaan. De vergoeding voor mijn deelname aan dit onderzoek zal in voorkomend geval aangepast kunnen worden.

Voor eventuele vragen, klachten, verdere opvolging, weet ik dat ik na mijn deelname terecht kan bij:

smec@kuleuven.be

Martine Verhees ([martine.verhees@ppw.kuleuven.be](mailto:simon.dewinter@ppw.kuleuven.be))

Prof. Kristina Casteels – pediater UZ Leuven ([kristina.casteels@uzleuven.be](mailto:kristina.casteels@uzleuven.be))

Onderzoek toont aan dat er geen sprake is van bijwerkingen na het gebruik van oxytocine of de placebo stof. Indien er tijdens of na het onderzoek toch nood is aan medische bijstand, dan is deze steeds beschikbaar.

Deze studie werd goedgekeurd door de Commissie voor Medische Ethiek van UZ KULeuven / Onderzoek.

Conform de Belgische wet van 7 mei 2004  inzake experimenten op de menselijke persoon, is de opdrachtgever zelfs foutloos, aansprakelijk voor alle schade die de deelnemer of zijn rechthebbenden opliepen en die rechtstreeks dan wel onrechtstreeks verband vertoont met het experiment . De opdrachtgever van deze studie [KULeuven] heeft een verzekering afgesloten die deze aansprakelijkheid dekt. Indien U schade zou oplopen ten gevolge van uw deelname aan deze studie zal die schade bijgevolg worden vergoed conform de Belgische wet van 7 mei 2004.

Datum:

Naam en handtekening proefpersoon: Naam en handtekening onderzoeker:
